# Transcription factors NF-YA2 and NF-YA10 regulate leaf growth via auxin signaling in *Arabidopsis*

**DOI:** 10.1038/s41598-017-01475-z

**Published:** 2017-05-03

**Authors:** Min Zhang, Xiaolong Hu, Ming Zhu, Miaoyun Xu, Lei Wang

**Affiliations:** 1 Biotechnology Research Institute/The National Key Facility for Crop Gene Resources and Genetic Improvement, Chinese Academy of Agricultural Sciences, Beijing, 100081 China; 2 School of Life Sciences, Anhui Agricultural University, Hefei, 230036 China

## Abstract

In plants, leaf is crucial for photosynthesis and respiration. Leaf area and quantity are important for leaf vegetables to increase biomass. The process of leaf development involves coordinated regulation among small RNAs, transcription factors and hormones. Here, we found leaf size were regulated by transcription factors NF-YA2 and NF-YA10 in *Arabidopsis*. *NF-YA2* and *NF-YA10* overexpression increased biomass accumulation through promoting leaf growth and cell expansion. *NF-YA2* and *NF-YA10* were expressed in SAM and leaf vasculature. Endogenous IAA content reduced by 20% and 24% in transgenic *Arabidopsis* plants overexpressing *NF-YA2* and *NF-YA10* compared to wild-type plants. Chromatin immunoprecipitation assays revealed that NF-YA2 and NF-YA10 bound directly to the *cis*-element CCAAT in the promoter of the *YUC2*, and decreased the expression of *YUC2*, a *YUCCA* family gene. The auxin transporter gene *PIN1* and auxin response factor1 and 2 (*ARF1* and *ARF2*) genes, transcriptional repressors, were downregulated. These findings showed leaf development was regulated by NF-YA2 and NF-YA10 through the auxin-signaling pathway and may provide a new insight into the genetic engineering of vegetables biomass and crop productivity.

## Introduction

Leaves are photosynthetic tissues and very important for the success of plants. The process of leaf development is composed of primordia initiation, lamina expansion and margin formation, involves coordinated regulation among small RNAs, transcription factors and hormones^1^. Genetic studies showed that many key factors involved in leaf development. MiR156/SPL regulation module has been reported to interact with TCP4 and this complex promoted CUC-controlled acquisition of leaf complexity in *Arabidopsis* ^2^. In relation to primordia initiation, miR160 targets *ARF10*, *ARF16*, and *ARF17*, three members of a divergent class of *ARF* genes that share high amino acid sequence similarity and present overlapping expression patterns^3–5^. The *ARF* genes regulated by miR160s are necessary for proper phyllotaxis in the rosette. Besides, MiR164 regulates organ boundary size through its modulation of the *CUC1* (CUP-SHAPED COTYLEDON1) and *CUC2* genes^5–7^. MiR319, also called miRJAW in *Arabidopsis*, is involved in the coordination of cell division and growth during leaf development by targeting a subset of the *TCP* (TEOSINTE BRANCHED/CYCLOIDEA/PROLIFERATING CELL FACTOR) genes that are homologues to the Antirrhinum *CIN* (CINCINNATA) gene^8^ and the tomato *LA* (LANCEOLATE) gene^9^. AT the gene level, *Class III HD-ZIP*, *KANADI* and *YABBY* gene families are involved in the establishment of polarity^10–12^. In addition, *PIN* and *CUC* genes play crucial role in leaf margin patterning by controlling auxin-maxima formation^13, 14^. Furthermore, *CIN* (CINCINNATA) gene limits excess cell proliferation and maintains the flatness of the leaf surface by directly modulating the hormone pathways involved in patterning cell proliferation and differentiation during leaf growth^15^.

Auxin is a key hormone that is responsible for modulating many aspects of plant growth, including root and leaf architecture, organ patterning, and vascular development^16^. Current models propose that members of the PIN protein family of auxin efflux regulators represent an important part of a network for auxin distribution throughout the plant^17^ and mediate auxin efflux from cells and thus directional cell-to-cell transport. *YUC* (YUCCA) family genes of *Arabidopsis* encode flavin monooxygenase-like enzymes that catalyze the rate-limiting step in Trp-dependent auxin biosynthesis^18^. *YUC* genes had been proved functions are important in leaf margin development and blade outgrowth^19^.

The miR169 family of *Arabidopsis* contains 14 genes. However, only four mature miR isoforms (a, b/c, d/e/f/g and h/i/j/k/l/m/n) are produced. The miR169 isoforms present distinct expression patterns during development^20^, in response to biotic^21^ or abiotic stresses^22, 23^, suggesting a functional specialization. In plants, the main targets of miR169 are genes that encode the subunit A of nuclear factor Y (NF-Y)^24^. This transcription factor (TF) is a heterotrimeric TF composed of NF-YA (HAP2), NF-YB (HAP3/CBF-A) and NF-YC (HAP5/CBF-C) subunits. In plants, NF-Y TFs have been linked to development^25–27^, signalization^28^ and responses to stresses^23, 29–31^. NF-YB and NF-YC subunits contain a histone fold domain very similar to H2A and H2B core histones^32, 33^ and these two subunits must form a heterodimer for stable interaction with NF-YA. The *NF-Y* genes present differential expression patterns during development^34–37^, or in response to environmental conditions^38, 39^, suggesting that, in different organs or under certain stimuli, only some combinations of subunits can be assembled to form the trimeric functional NF-Y factor. *Arabidopsis* miR169d/NF-YA2 (10) modules had been clearly shown that it plays a crucial role in stress-induced early flowering^40^ and root architecture in *Arabidopsis* ^41^.

Here, we showed that NF-YA2 (10) plays important roles in leaf development. Our data suggested that NF-YA2/10 can directly interact with *YUC2* promoter, and decreased *YUC2* expression, which in turn regulates the synthesis of auxin.

## Results

### *NF-YA2* and *NF-YA10* overexpression promote leaf initiation and development

**Figure 1:**
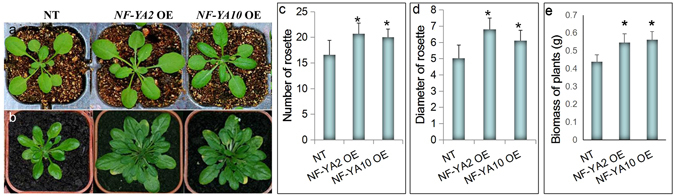
Rosette phenotype of *NF-YA2* and *NF-YA10* overexpression lines. (**a**) 20-days-seedling; (**b**) plants at bolting stage; (**c**) number and diameter of rosettes for *NF-YA2* and *NF-YA10* overexpression lines. Thirty plants were measured for each line.

We observed that overexpression of *NY-YA2* was not only regulated *Arabidopsis* flowering time, but also affected leaf development. To illustrate the potential role of *NY-YA2* and *NY-YA10* in leaf development, we constructed *NF-YA10* overexpression vector and obtained the transgenic plants. In contrast to NT, *NF-YA2* and *NF-YA10* overexpression plants showed larger rosettes (Fig. 1a). The rosette diameter of *NF-YA2* OE and *NF-YA10* OE plants were 6.8 and 6.5 cm, respectively, larger than NT (5.03 cm) (Fig. 1d). Moreover, *NF-YA2* and *NF-YA10* overexpression plants can generate new rosette leaves incessantly even after seeds harvest, whereas the leaves of NT plants generally decayed after harvest (Fig. 1b). Thereof *NF-YA2* OE and *NF-YA10* OE plants can generated more leaves than that in NT. The 40-days rosette numbers of NT, *NF-YA2* OE and *NF-YA10* OE were 16.6, 20.7 and 20, respectively (Fig. 1c). The biomass of *NF-YA2* OE and *NF-YA10* OE were increased by 24% and 28% compared to NT (Fig. 1e).

### *NF-YA2* and *NF-YA10* overexpression expand cell size of leaves

**Figure 2:**
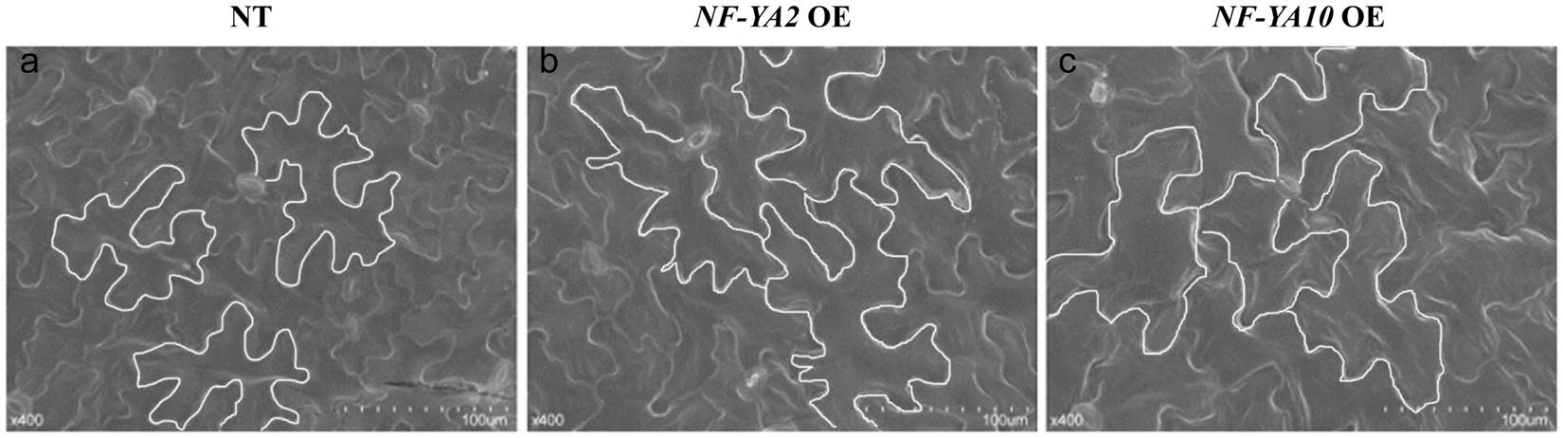
SE micrograph of a leaf of 8-week-old plant. (**a**) Negative transgenic plant; (**b**) *NF-YA2* OE plant; (**c**) *NF-YA10* OE plant.

The leaf size is determined generally by cell number and cell size. To uncover what reason result in larger leaf in *NY-YA2* OE and *NF-YA10* OE plants, we investigated their cell size and numbers using scanning electron (SE) microscopy. The epidermal cells of the leaves in *NF-YA2* and *NF-YA10* OE plants were larger than those in NT (Fig. 2a–c). These results indicate that NF-YA2 and NF-YA10 regulate leaf size by controlling cell size.

### *NF-YA2* and *NF-YA10* are expressed in shoot apical meristems, internode and leaves

**Figure 3:**
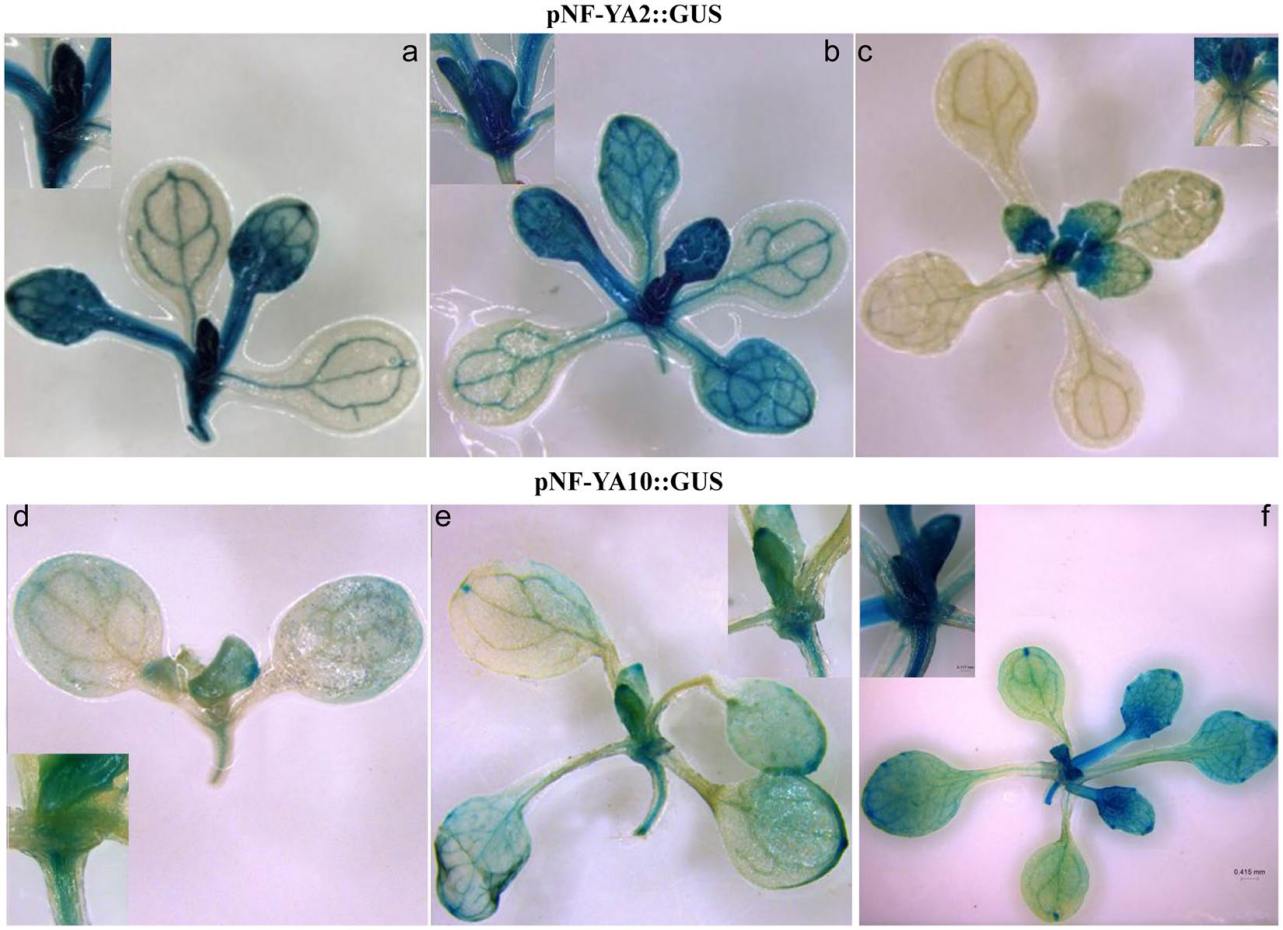
Expression profile of *NF-YA2* and *NF-YA10* in transgenic plants. (**a**–**c**) *NF-YA2* expression pattern in 10-d-old, 13-d-old, and 16-d-old seedlings; (**d**,**e**) *NF-YA10* expression pattern in 10-d-old, 13-d-old, and 16-d-old seedlings. Plants were grown on MS medium in a standard LD light regime.

In order to explore the expression patterns of *NF-YA2* and *NFYA10*, WT plants were transformed with promoter::GUS constructs that contained the 2 kb promoter region from *NF-YA2* and *NF-YA10* respectively. *NF-YA2* and *NF-YA10* have high level expression patterns in cotyledon vasculature and SAM in *pNF-YA2::GUS* and *pNF-YA10::GUS* plants, suggesting that they might have a role in leaf initiation and developing. *NF-YA2* was expressed mainly in SAM, node and young leaves, and the expression level was rapidly decreased with leaf growth (Fig. 3a–c). *NF-YA10* was expressed in SAM, node and leaves, and the expression level was increased with leaf growth. However the expression level of *NF-YA10* was clearly weaker in SAM and node than that of *NF-YA2* (Fig. 3d–f). The highest expression level of *NF-YA2* and *NF-YA10* was in SAM region and young leaves. Considering auxin is synthesized mostly in SAM and young leaf, we reduced that they might be involved in IAA regulation to affect leaf initiation and development.

### *NF-YA2* and *NF-YA10* overexpression decreased endogenous IAA content

**Figure 4:**
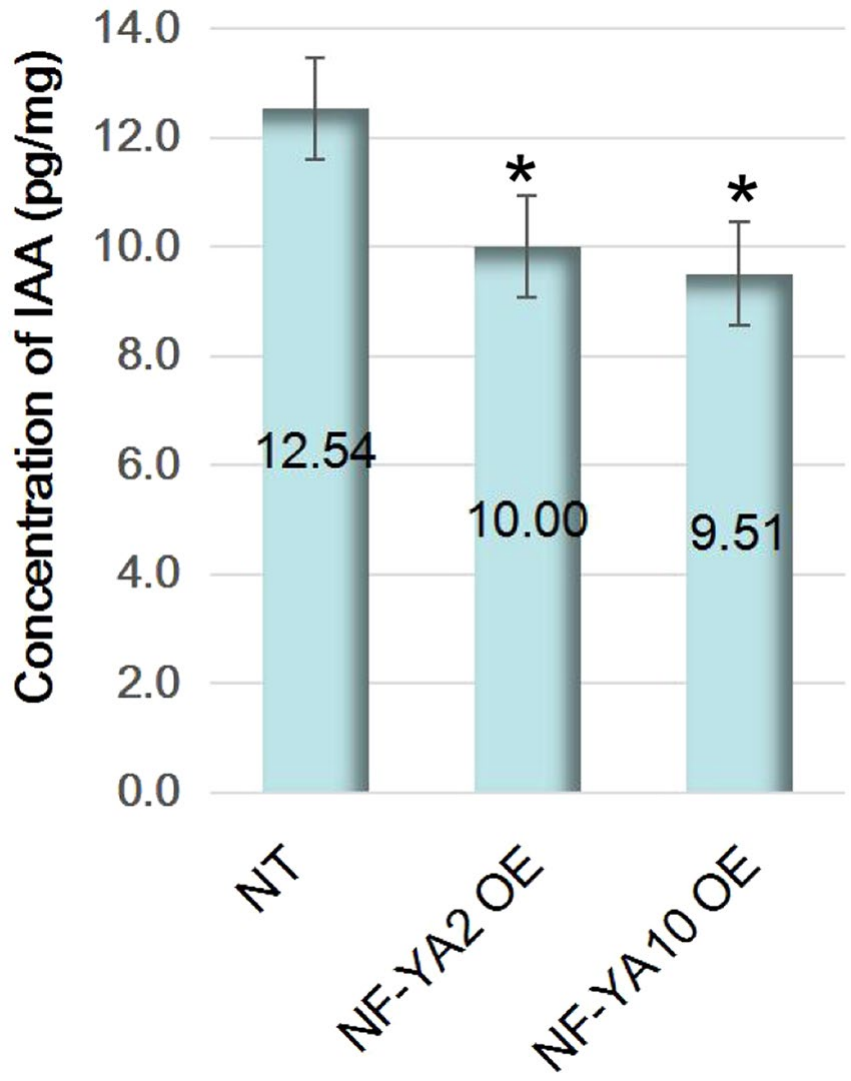
Concentrations of IAA in transgenic plants and NT.

Auxin has been confirmed as the central regulator of organogenesis at the SAM. Considering the SAM expression profiles of *NF-YA2* and *NF-YA10*, we investigated concentration of endogenous IAA in whole shoots including rosette and SAM. The data showed that IAA concentrations were decreased by 20% and 24% in the *NF-YA2* and *NF-YA10* overexpression lines, respectively, compared to the NT (Fig. 4), indicating *NF-YA2* and *NF-YA10* are involved in regulation of IAA biosynthesis and auxin-signaling pathway.

### *NF-YA2* and *NF-YA10* are involved in regulation of auxin biosynthesis

**Figure 5:**
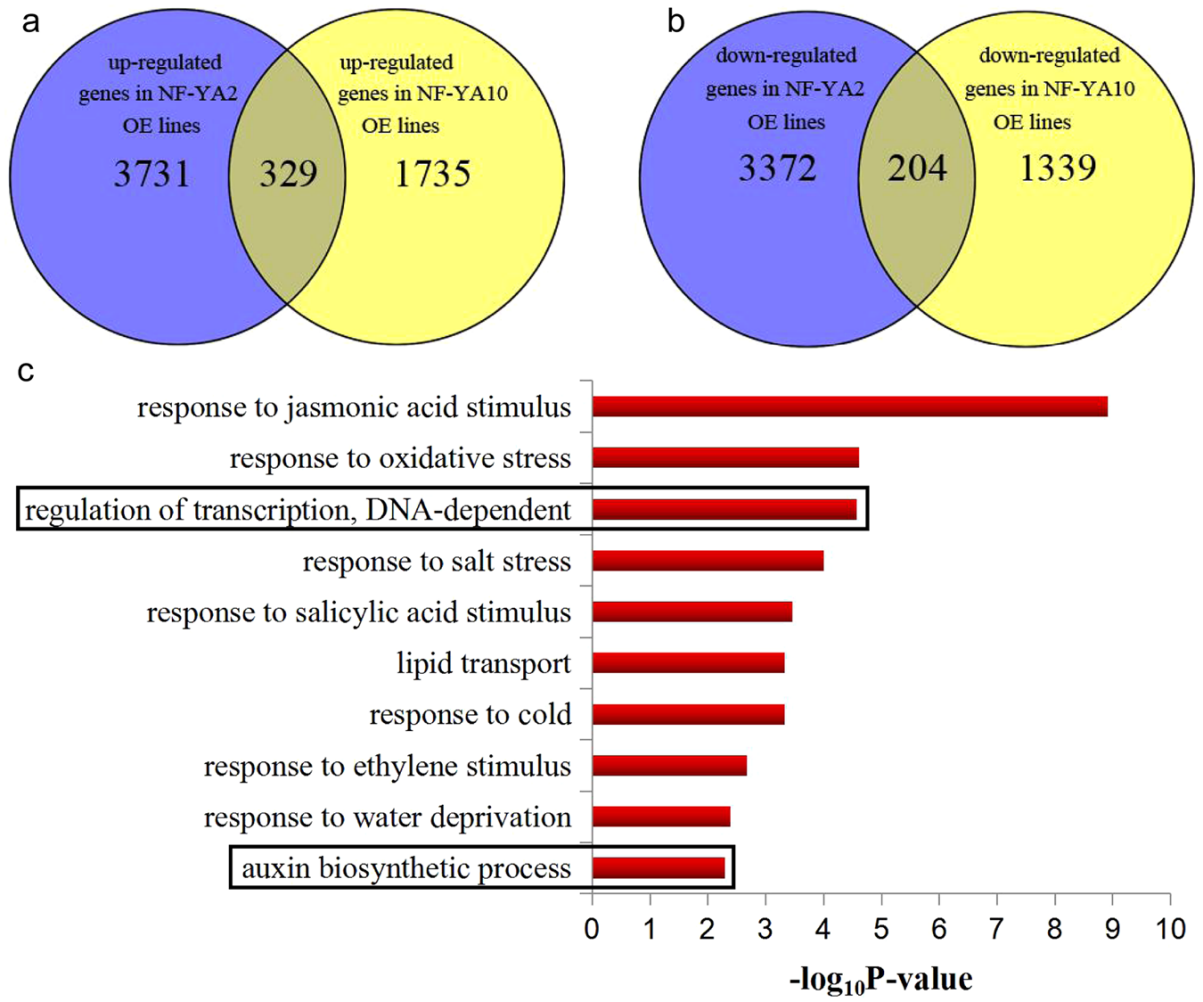
Up (**a**) and down (**b**) DEGs between *NF-YA2* OE plant and *NF-YA10* OE were analyzed using VENN and significantly enriched Gene Ontology (GO) categories with the common DEGs (**c**).

To understand the molecular mechanisms and genetic regulation of leaf generation and development, we profiled the transcriptomes of developing node and leaves from *NF-YA2* OE and *NF-YA10* OE plants, respectively. Total of 7631 and 3607 differentially expressed genes (DEGs) were identified using cut-off values (log2FC >1 or <−1 with *p*-value of 0.05) in comparison with NT (Fig. 5a,b). GO enrichment analysis was performed on these two groups of DEGs to discover overrepresented functional categories (Fig. 5c). Top 10 generally changed GO terms by enrichment score (−log10P-value) were showed in Fig. 5c. The most enriched and meaningful biological process terms were related to stress responding, regulation of transcription, and plant hormone signaling, suggesting NF-YA transcription factors in plants are potentially involved in stress responding and plant development. Based on alerted endogenous IAA content in AtNF-YA2 and AtNF-YA10 OE plants, we examined differential accumulation of auxin signaling, such as auxin biosynthetic process (GO:0009851), and found that the expression of YUCCA family was clearly different between transgenic plants and NT. YUCCA family members of *Arabidopsis* encode Flavin monooxygenase-like enzymes that catalyze the rate-limiting step in Trp-dependent auxin biosynthesis, which play important roles in local auxin biosynthesis^18^. The YUC is known as a key factor in the regulatory pathway controlling leaf development^42^. YUC-controlled leaf developmental pathway acts synergistically with auxin polar transport^43^. Three genes *YUC1*, *YUC2*, and *YUC6* were down-regulated in *NF-YA2* OE and *NF-YA10* OE lines (Supplementary Table S1). Together, functional characterization of DEGs between *NF-YA2* OE line and *NF-YA10* OE line indicated that NF-YA2 (10) maybe regulate auxin biosynthesis via *YUCCA* family genes.

**Figure 6:**
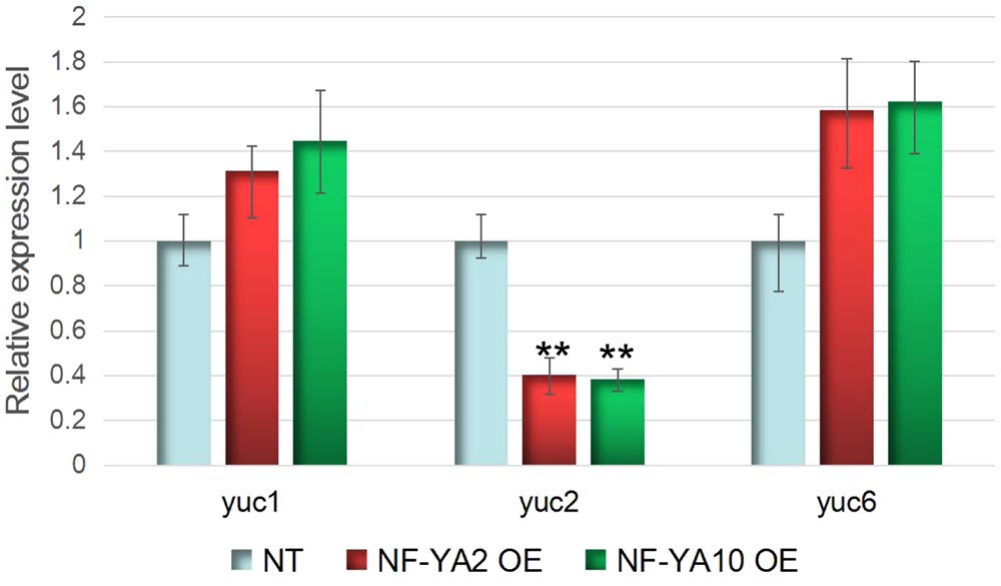
*Yuc1, yuc2* and *yuc6* expression in NT, *NF-YA2 OE* and *NF-YA10* OE lines.

Based on the transcriptomes analysis, *YUCCA* family genes (*YUC1*, *YUC 2*, and *YUC6*) were potential targets regulated by NF-YA2 and NF-YA10. Thereof we investigated their expression in NT plants, *NF-YA2* OE and *NF-YA10* OE lines. *YUC 1* and *YUC6* expression in *NF-YA2* OE and *NF-YA10* OE plants were higher than in NT, but their differential expression were not significant. However, *YUC2* was significantly down-regulated in the *NF-YA2* OE and *NF-YA10* OE plants (Fig. 6). These results suggest NF-YA2 and NF-YA10 can specifically regulate *YUC2* expression.

### NF-YA2 and NF-YA10 specifically regulate *YUC2* expression

**Figure 7:**
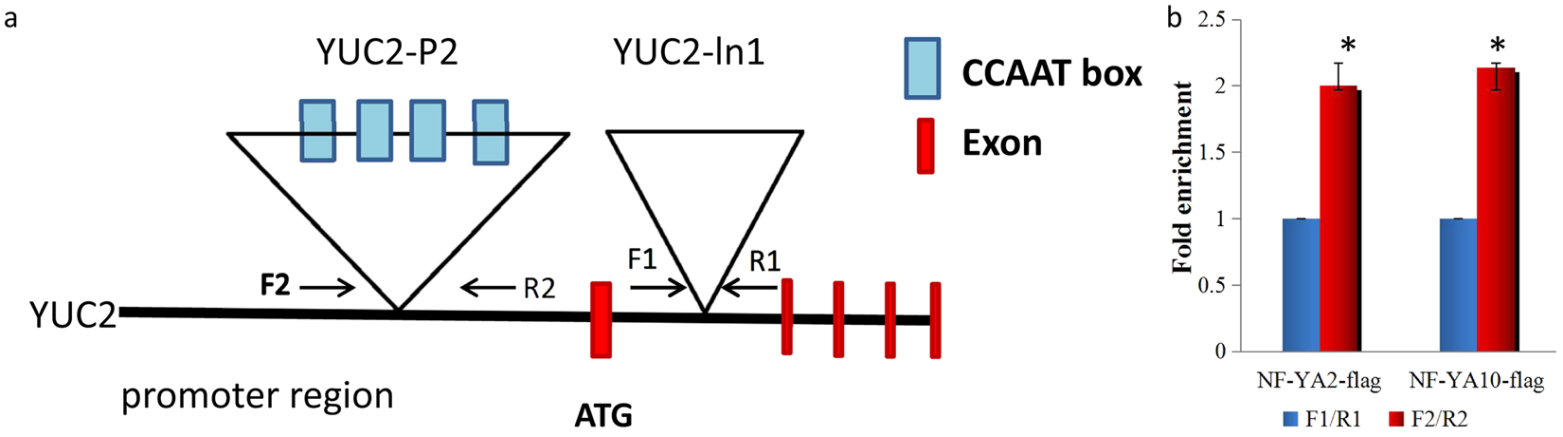
Chromatin immunoprecipitation (ChIP) assay in *NF-YA2-FLAG* and *NF-YA10-FLAG* plants. (**a**) Schematic structure of genomic sequences of yuc2 and the regions examined by ChIP. Two pairs of primers were used. Primer YUC2-P2 covered the promoter region containing the CCAAT *cis*-element. (**b**) Relative levels of qPCR products from the ChIP assay. Data were from one experiment with three technical replicates. Values are means ± SD, n = 3.

Previous studies in human, animals and plants suggested that the CCAAT box is a binding site for NF-YA protein^44, 45^. Sequence analysis suggests that there are several CCAAT motifs located in the *yuc2* promoter and the first intron (Fig. 7a). To investigate if NF-YAs bind to these sites, FLAG-tagged *NF-YA2* and *NF-YA10* transgenic *Arabidopsis* plants were obtained^45^, and ChIP was performed on the transgenic plants using anti-FLAG antibodies. Quantitative PCR (qPCR) was then performed on the *YUC2* sequences using four different pairs of primers (Fig. 7a). As shown in Fig. 7b, the induced *NF-YA2* and *NF-YA10* transgenic plants showed a clear enrichment of the promoter (YUC2-P1and YUC2-P2) sequences in comparison with mock transgenic *Arabidopsis*. These results suggest that NF-YA2 and NF-YA10 regulates the expression of *YUC2* by physically interacting with *YUC2* promoter.

### Effect of *NF-YA2 and NF-YA10* on the expression of genes involved in IAA signaling pathway

**Figure 8:**
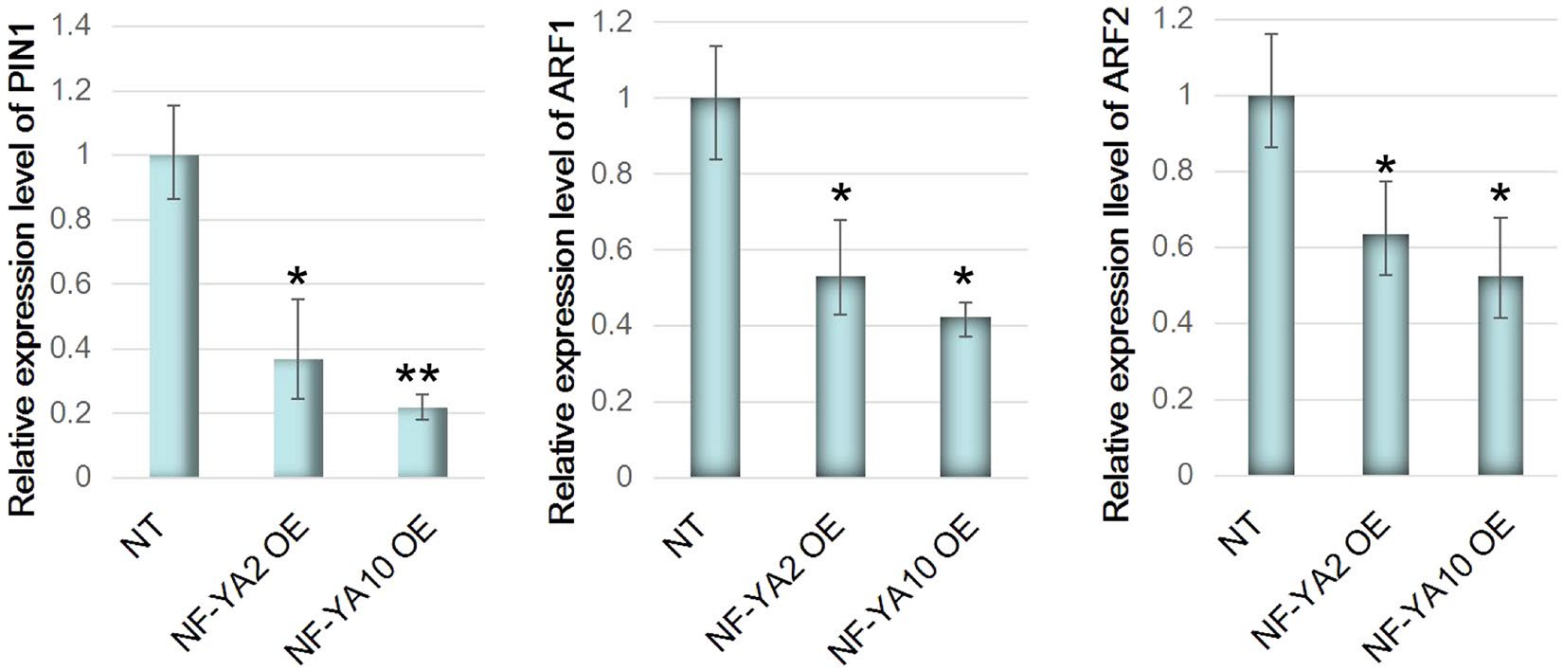
Relative expression levels of *PIN1*, *ARF1* and *ARF2* in *NF-YA2* OE and *NF-YA10* OE plants.

Based on the endogenous IAA contents were decreased in the overexpression plants, the auxin transport and signaling should be changed accordingly. Thereof we examined the expression levels of the IAA efflux carrier protein gene *PIN1* and auxin response factor gene *ARF1*. The data showed *PIN1* and *ARF1* expression was decreased significantly in *NF-YA2OE* and *NF-YA10OE plants* compared to NT plants (Fig. 8). These results indicated that the expression of *PIN1, an* IAA transporter, was decreased with lessened IAA concentration. *ARF1*, a repressor of auxin-induced genes, can be bound and repressed by Aux/IAA protein at low IAA concentration, delaying the process of aging (leaf increasing) in *Arabidopsis*. Accordingly, downregulated expression of *ARFs* in *NF-YA2* OE and *NF-YA10* OE plants also presented leaf increasing and preventing senescence. We deduced that low IAA might result in more and larger rosettes through ARF family.

## Discussion

### *NF-YA2* and *NF-YA10* mediated leaf development

Leaf primordia of higher plants derive from the peripheral zone of the shoot apical meristem. Major outstanding questions in leaf development are initiation of the primordia, leaf patterning and ending, and how these processes are regulated accurately plays an important role in the plant life. Transcription factors are known act as regulation hub to play crucial roles in plant development processes and in response to environmental and endogenous conditions, however few of them have been linked to leaf growth. Here, we found that *NF-YA2* and *NF-YA10* genes were involved in leaf initiation and growth.

Members of *NF-YA* family can be regulated by miR169 family, which are involved in drought and nitrate responses, flowering and root architecture. Overexpression of the *miR169a*, which specifically targets *NF-YA5*, induced drought sensitivity or altered nitrogen responses^23, 30^ and miR169d-g mature sequence is induced by nitrate deficiency^46^. *NF-YA2*, targeted by *miR169d*, was involved in stress-induced flowering in *Arabidopsis* ^40^. Moreover, the regulation modules of *miR169d/e/f/g* isoform and the *NF-YA2* target control root architecture in *Arabidopsis* ^41^. Recently, in roots, a clear upregulation of *NF-YA2* and *NF-YA10* has been observed in response to phosphate starvation^47^. Our data showed that NF-YA2 and NF-YA10 were involved in leaf development via regulating IAA biosynthesis. These results, all together, are consistent with the fact that the miR169/NF-YA module, could directly or indirectly act as a linker between plant development and responding to abiotic stresses. Indeed, auxin is a well-known operators of growth and development, which can be affected by all of these stresses in plants^48^.

**Figure 9:**
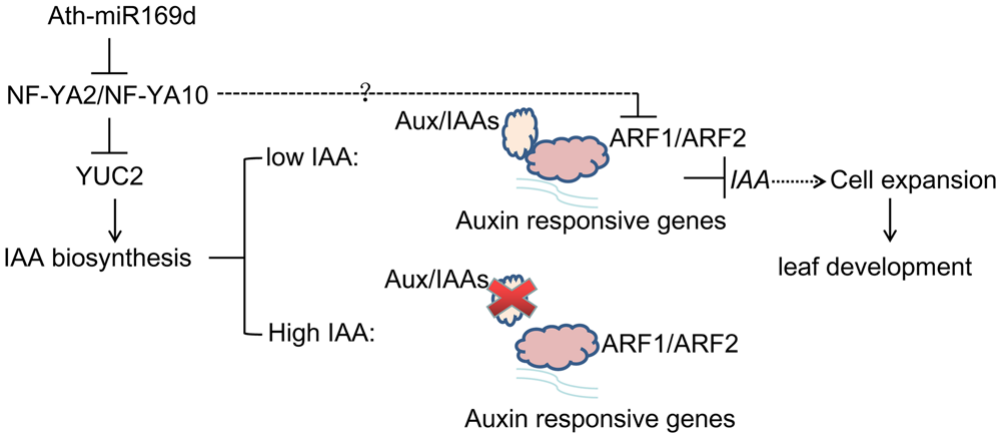
Schematic model of NF-YA2 and NF-YA10 mediated regulation of *Arabidopsis* leaf initiation and development through auxin signaling. IAA: indole-3-acetic acid; YUC2: YUCCA 2; ARF1: auxin response factor 1.

Based on the previous results and our finds, we put forward a model showed in Fig. 9. *YUC2*, a key speed-limiting gene in auxin homeostasis, acts as a direct target of NF-YA2 and NF-YA10. Overexpression of *NF-YA2* and *NF-YA10* decreased contents of endogenous IAA through repressing *yuc2* expression. Lower IAA contents result in downregulation of *PIN* and *ARFs* family. *ARF1* and *ARF2*, transcriptional repressors, can directly bind to promoters that contain auxin response elements (TGTCTC) to repress targets transcription, such as *IAA*, further influence leaf initiation and growth in *Arabidopsis* (Fig. 9).

Over all, NF-YA family members have been proposed to control various plant responses to environmental stresses^49^ and development^43^. Our data showed that NF-YA2 and NF-YA10 were involved in leaf growth in *Arabidopsis* through IAA biosynthesis, providing a new insight for *miR169/NF-YA* module roles between abiotic stress and development.

### Auxin and leaf development

All plant shoots can be described as a series of developmental modules termed phytomers, which are produced from SAM. A phytomer generally consists of a leaf, internode, and a secondary shoot meristem. Because leaf formation is part of the general lateral organ initiation program at the SAM, it is not surprising that auxin is involved. Classical micromanipulation techniques and probes that predict auxin transport pathways confirmed that dynamic auxin fluxes pattern organ initiation at the shoot apex, suggesting that auxin plays a critical role in leaf development^50^. Leaf initiation and leaf growth are different progress, leaf initiation requires the formation of an auxin maximum and leaf growth needs transcriptional responses mediated by ARFs^51^. Here concentration of endogenous IAA in whole shoots pooled rosette and SAM was decreased, which seemed to be conflicting to leaf initiation promotion. Because the amount of SAM was negligible compared to whole rosette, SAM should be separated to detect the concentration of endogenous IAA in the further experiment.

The phenotypic similarities in leaves between the *NF-YA2* OE or *NF-YA10* OE plants and the *arf1* and *arf2* mutants^52^ support the notion that the *NF-YA* genes affect an auxin-signaling process. Meanwhile certain *yuc* mutants were treated by combination with the auxin transport inhibitor NPA totally blocked new leaf formation, a phenotype that is not observed in the *yuc* mutants alone or NPA treatment alone^42^, suggesting that leaf development is regulated by coordinated auxin biosynthesis, transport and signalling response. So we presumed that NF-YA2 or NF-YA10 maybe target other genes in auxin-signaling pathway besides *yuc2*, which should be analyzed in the future.

## Methods

### Plant materials and culture

All experiments were performed on the Columbia ecotype of *A. thaliana*. Plants were grown in a controlled culture room at 22 °C with a relative humidity of 60% and 16/8 h photoperiod.

### Constructs and transgenic lines

For pNF-YA2::GUS and pNF-YA10::GUS, 2000-bp region upstream of the start codon ATG of NF-YA2 and NF-YA10 was amplified from genomic DNA (all primers sequences used for cloning are listed in Table S2), respectively, to cloned into pEASY-T1 vector, which were recombined in the binary vector pCAMBIA1303 after sequencing confirmation.

The p35S::NF-YA2 overexpression lines and transgenic NF-YA2-flag plants had been generated by Xu *et al*.^40^.The p35S::NF-YA10 and NF-YA10-FLAG constructs were obtained as described^40^. Col 0 transformation was performed by the floral dip method^53^ and independent stable transgenic lines were selected.

### GUS staining and microscopy

The histochemical detection of GUS activity was performed as described^54^ with a staining incubation overnight. Then the stained tissues were decolorized by 75% ethanol and the images were obtained using a microscope. To analyze expression in the whole organ, seedlings with different stage were obtained to detect.

### RNA extraction, Real time fluorescence quantitative PCR (qRT-PCR)

Total RNA was extracted by using the Trizol procedure as described by the manufacturer (ambion) and cDNA was synthesized following the manufacturer’s instructions (5x All-In-One RT MasterMix, abm, Canada). qRT-PCR (all qPCR primers sequences used can be found in Table S2) was performed on an Applied Biosystems (http://www.AppliedBiosystems.com) Prism 7500 analyzer and SYBR Premix Ex Taq™ (CodeQPK-201, TOYOBO). For each genotype, three or four independent biological replicates, each consisting of 10 individual plants, were analysed. Sample comparisons were performed using the 2(−ΔΔCT) method^55^. *Actin1* was reference control.

β-Oestradiol treatment of transgenic NF-YA2-FLAG and NF-YA10-FLAG plants and ChIP(Chromatin immunoprecipitation) assay were carried out as described^40^. All the primers used for ChIP-qPCR are listed in Supplementary Table S2.

### Microarray analysis

Total RNA was extracted from seedlings of *NF-YA2* OE and *NF-YA10* OE lines. RNA quantity and quality were measured by Agilent 2100 Bioanalyzer. RNA integrity was assessed by standard denaturing agarose gel electrophoresis. Agilent *Arabidopsis* Oligo Microarray V4.0 was adopted for detection of mRNA expression. All the microarray analysis was performed by oe Bio-tech (Shanghai, China).

### Quantitative analysis of IAA

Whole shoots were harvested from plants grown under a 16:8-h photoperiod in trays (12 seedlings per tray) when the first open flower was visible. Each of the randomly arranged trays contained a single genotype and represented one replicate sample. Three replicate samples (200 mg fresh weight) were analyzed. Total IAA were detected and quantified as methyl esters by gas chromatography–mass spectroscopy (GC–MS) at Institute of Genetics and Development Biology, Chinese Academy of Sciences. (Beijing, China).

## Electronic supplementary material

title page and Table S1 S2
