# Supplementary material for: Transcription factors NF-YA2 and NF-YA10 regulate leaf growth via auxin signaling in Arabidopsis
Source: Sci Rep. 2017 May 3;7:1395. doi: 10.1038/s41598-017-01475-z (PMC5431230; doi:10.1038/s41598-017-01475-z)
Supplement: Supplementary file 1 — title page and Table S1 S2 [file 41598_2017_1475_MOESM1_ESM.pdf]

Title：

Transcription factors NF-YA2 and NF-YA10 regulate leaf growth via auxin signaling in *Arabidopsis*

Author：

1. Min Zhang[1]， Email：13126532159@163.com
2. Xiaolong Hu[1]，Email：sw0401@sjtu.edu.cn
3. Ming Zhu[1，2]，Email：lyanzhu0@126.com
4. Miaoyun Xu[1]*，Email：xumiaoyun@caas.cn
5. Lei Wang[1]*，Email：wanglei01@caas.cn

1. Biotechnology Research Institute/The National Key Facility for Crop Gene Resources and Genetic Improvement, Chinese Academy of Agricultural Sciences, Beijing, 100081, China
2. School of Life Sciences, Anhui Agricultural University, Hefei, 230036, China

*corresponding. xumiaoyun@caas.cn and wanglei01@caas.cn

Table S1. Expression level of *YUC* genes in *NF-YA2* and *NF-YA10* overexpression lines

Gene	Log FC (NF-YA2 OE vs WT)	Log FC (NF-YA10 OE vs WT)	Regulation	Gene Name	Genomic location	GO
AT4G32540	-1.486	-0.2443223	down	(*YUC1*) YUCCA family monooxygenase	chr4:15702797-15702856	GO:2000024|GO:0050661|GO:0048827|GO:0048825|GO:0009911|GO:0004499|GO:0050660|GO:0022603|GO:0016491|GO:0009851|GO:0010229
AT4G13260	-0.721	-0.6713195	down	(*YUC2*)Flavin-binding monooxygenase family protein	chr4:7721908-7721849	GO:0050661|GO:0004499|GO:0050660|GO:0016491|GO:0009851
AT5G25620	-0.691	-1.03258	down	(*YUC6*)flavin-containing monooxygenase-like protein	chr5:8935595-8935536	GO:0050661|GO:0004497|GO:0004499|GO:0050660|GO:0016491|GO:0009851

Table S2. Primers Sequences for PCR

	Oligo Name	Primer sequence(5'to3')
For promoter cloning	pNF-YA2-FW	GGAAGCTTCCATAATAATCAAGTCC
	pNF-YA2-RV	CTCCAAATTCCAATTACAAAAAG
	pNF-YA10-FW	AATCGAAAGAGTTCACCTCGCC
	pNF-YA10-RV	GGCTAAGGATCACTTGGAATTG
For qRT-PCR For CHIP	YUC2-F1	CCATGATCCGTACGTGGAGG
	YUC2-R1	GACGCTATGCAAGTGGAACG
	YUC2-F2	GTGACACGGATCGGTTAGGG
	YUC2-R2	GCCAACGTCCAAAACAGGAG
	actin2-FW	TCCCTCAGCACATTCCAGCA
	actin2-RV	GATCCCATTCATAAAACCCCAGC
	AT-PIN1 qPCR F1	GCTATGATCCTCGCTTACGG
	AT-PIN1 qPCR R1	CGGAGGTTCATGGCGTAAGG
	ARF1-qPCR F1	GCTCGCTTTGGATCCAGTGG
	ARF1-qPCR R1	AGAGGTCCAGCACAGGCATG
	ARF1-qPCR F2	TGGATCAAAGCGAACCCACT
	ARF1-qPCR R2	TCCGTAGCACCGAGAAGCCA
	ARF2-qPCR F1	CGCTCGTCGTCGGTATTCAAG
	ARF2-qPCR-R1	ACTCGCTTCGGTAGTCCACT
	ARF2-qPCR F2	CTTCCTCCACCTCCGAGGTT
	ARF2-qPCR R2	AGTGGGAGGCTGTCGAGACA
	YUCCHIP-F1	CTATAGCATTAGACAAAAAC
	YUCCHIP-R1	GAGGATGAACAAAACTCTTC
	YUCCHIP-F2	GAAGAGGTTTTGCGGTTCTG
	YUCCHIP-R2	GTTCAACTAGCTATACACCG
